# Carta ao Editor Referente às Diretrizes Brasileiras de Hipertensão Arterial – 2020

**DOI:** 10.36660/abc.20210873

**Published:** 2022-07-07

**Authors:** Leticia Costa Rebello, Marcos Christiano Lange, Rodrigo Bazan, Maramelia Miranda Alves, Gisele Sampaio Silva, Octavio Pontes

**Affiliations:** 1 Hospital de Base do Distrito Federal Brasília DF Brasil Hospital de Base do Distrito Federal, Brasília, DF – Brasil; 2 Universidade Federal do Paraná Curitiba PR Brasil Universidade Federal do Paraná, Curitiba, PR – Brasil; 3 Universidade Estadual Paulista Júlio de Mesquita Filho Faculdade de Medicina Campus de Botucatu Botucatu SP Brasil Universidade Estadual Paulista Júlio de Mesquita Filho – Faculdade de Medicina Campus de Botucatu, Botucatu, SP – Brasil; 4 Universidade Federal de São Paulo Escola Paulista de Medicina São Paulo SP Brasil Universidade Federal de São Paulo - Escola Paulista de Medicina, São Paulo, SP – Brasil; 5 Universidade de São Paulo Hospital das Clinicas da Faculdade de Medicina de Ribeirão Preto Ribeirão Preto SP Brasil Universidade de São Paulo - Hospital das Clinicas da Faculdade de Medicina de Ribeirão Preto, Ribeirão Preto, SP – Brasil

**Keywords:** Hipertensão, Acidente Vascular Cerebral Hemorrágico, Acidente Vascular Cerebral Isquêmico, Fatores de Risco, Mortalidade, Tratamento, Pressão Arterial

**Prezado editor,**

Inicialmente, a Sociedade Brasileira de Doenças Cerebrovasculares (SBDCV) parabeniza o Departamento de Hipertensão Arterial da Sociedade Brasileira de Cardiologia, a Sociedade Brasileira de Hipertensão e a Sociedade Brasileira de Nefrologia pela realização das Diretrizes Brasileiras de Hipertensão Arterial 2020.^1^ Gostaríamos, no entanto, de tecer algumas considerações relacionadas ao cuidado dirigido ao paciente com acidente vascular cerebral (AVC) agudo. O texto atribui corretamente a Hipertensão Arterial como principal causa de AVC isquêmico (AVCi) e AVC hemorrágico (AVCh). Apontaremos, a seguir, algumas considerações quanto ao manejo da pressão arterial (PA) nesses pacientes.

Em relação ao controle da PA do paciente com AVCh, foi mencionado na diretriz, no item 10.6.1, que “estudos robustos sugerem que reduzir a PA (dentro de 6h) para valores <140/90mmHg não diminui eventos primários importantes, inclusive mortalidade”, com referência ao estudo INTERACT-2.^2^ A elevação da PA na fase aguda, resposta fisiológica ao quadro de AVCh, correlaciona-se a um pior prognóstico e expansão do hematoma, dado demonstrado no estudo INTERACT-1.^3^ Posteriormente, o estudo de fase 3 INTERACT-2 avaliou o controle intensivo de PA nestes pacientes, com meta Pressão Arterial Sistólica (PAS) <140mmHg *versus* controle de diretrizes da época (meta de PAS <180mmHg). Analisando o desfecho primário de morte ou dependência funcional ( *modified Rankin Scale* – mRS: 0-3 versus 4-6), não foi alcançada significância estatística em relação à redução de PA na fase aguda para esse desfecho (55.6% no tratamento convencional *versus* 52% com tratamento agressivo da PA, p=0,06).^2^ O desfecho secundário analisado, padrão de distribuição *shift* na mRS, também não teve resultados estatisticamente significativos. Entretanto, quando avaliado em análise ordinal, houve menor incapacidade (mRS 0-2) no grupo com tratamento intensivo da PA, com *odds ratio* 0.87 (IC 95%, 0,77-1,00; p=0,04), além de melhor qualidade física e mental, medidos pela escala EQ-5D, no grupo tratado com o controle intensivo da PA.^2^ Ao contrário do que a atual Diretriz Brasileira de Hipertensão Arterial recomenda, e baseada nos resultados dessa análise ordinal, a recomendação atual da *American Heart* e *American Stroke Association,* ^4^ endossada pela SBDCV, é a redução aguda da PAS em pacientes com AVCh que se apresentem com PAS elevada entre 150-220mmHg e que não tenham contraindicações ao controle intensivo da PA, com alvo PAS <140mmHg, medida que pode ser efetiva na melhora do desfecho clínico funcional. Ainda não há dados suficientes para sustentar de maneira sistemática a segurança e a efetividade do manejo agudo de PA em pacientes com PAS >220mmHg, porém considerase razoável a redução agressiva da PA nesse perfil de pacientes, com infusão de medicamentos endovenosos com titulação de dose e controle rigoroso da PA na fase aguda.^4^ Com base nos dados do INTERACT-2 e na recomendação das sociedades supracitadas, gostaríamos de sugerir a correção dos itens 10.6.1 e 13.7.2 na diretriz em questão, quando se aponta ausência de benefício em redução de incapacidade grave com o controle intensivo da PA.^1^ Reforçamos ainda que, ao contrário do exposto no texto, já foi também demonstrado em estudos clínicos que a redução proposta da PA é segura.^2 – 4^ A SBDCV não recomenda o alvo proposto de PAS <180mmHg para o AVCh apontado na diretriz.

Em relação ao manejo de PA no AVCi agudo, tópico 10.6.2, reforçamos que a redução da PA em pacientes candidatos a trombólise deverá ser feita quando os valores estiverem >185/110mmHg na primeira hora. Após o término da trombólise, de fato, o valor de PA recomendado é <180/105mmHg nas primeiras 24 horas, como apontado na diretriz de Hipertensão.^1^

Em relação ao tópico 13.7.1, a PA recomendada para indicação de tratamento trombolítico é <185/110mmHg, devendo ser iniciado medicamento anti-hipertensivo endovenoso imediatamente, para tentativa de correção, caso a medida inicial de PA esteja acima desse patamar. A contraindicação à trombólise ocorre somente se houver PA refratariamente elevada em três medidas consecutivas, com intervalo de 5 minutos entre elas, a despeito de tratamento otimizado.

Também desconhecemos a referência que sugere redução imediata da pressão arterial em pacientes com ataque isquêmico transitório, conforme sugerido no quadro 10.2.^1^ Ao contrário, o AIT é considerado um evento equivalente ao AVCi agudo, e deve ser manejado com os mesmos parâmetros do AVCi não trombolisado ou não tratado com trombectomia mecânica, ou seja, tolerabilidade de PA até 220/120mmHg, usualmente com a suspensão de drogas antihipertensivas por via oral na fase hiperaguda do atendimento, e desde que não existam outras condições cardiovasculares impeditivas para permitir estes níveis pressóricos (por exemplo, infarto agudo do miocárdio, aneurisma ou dissecção de aorta).^5^ Assim, cabe também uma revisão do tópico e quadro sobre as recomendações de redução imediata da PA no AIT, e de não redução da PA em AVCi de uma forma geral (quadro 10.2).

É sabido que a denominação do AVC no nosso país é bastante diversa, até mesmo entre médicos, a depender do estado ou região do país (uso de termos “AVC - Acidente Vascular Cerebral” e “AVE - Acidente Vascular Encefálico”), fato demonstrado inclusive em estudo brasileiro sobre este tema.^6^ Por este motivo, nos últimos anos, a grande maioria dos especialistas em Neurologia Vascular e organizações de pacientes, em conjunto com a SBDCV e o Departamento Científico de Doenças Cerebrovasculares da Academia Brasileira de Neurologia, têm recomendado e disseminado, tanto no meio acadêmico quanto em materiais e campanhas educativas junto à população, comunicados, entrevistas na mídia e redes sociais, a unificação do termo ideal na nossa língua como sendo a sigla “AVC”, visando uma maior educação da população quanto à doença, e evitando-se o uso de outros termos, fator que pode confundir e dificultar o seu reconhecimento rápido, primordial ao adequado tratamento e melhor prognóstico. Assim, sugerimos, em documento futuro da Diretriz de Hipertensão, rever a utilização do termo “AVE”, preferindo-se denominar a doença como temos denominado e advogado nos últimos anos: Acidente Vascular Cerebral – AVC.

Por fim, agradecemos a oportunidade desta manifestação, e em nome da Sociedade Brasileira de Doenças Cerebrovasculares e do Departamento Científico de Doenças Cerebrovasculares da Academia Brasileira de Neurologia, nos colocamos à disposição para futuras colaborações e discussões de tópicos que envolvam o manejo de pacientes com AVC.
