# Supplementary material for: Carta ao Editor Referente às Diretrizes Brasileiras de Hipertensão Arterial – 2020
Source: Arq Bras Cardiol. 2022 Jul 7;119(1):139–42. [Article in Portuguese] doi: 10.36660/abc.20210873 (PMC9352129; doi:10.36660/abc.20210873)
Supplement: Translation

# Translation: Letter to the Editor Regarding the Brazilian Guidelines of Hypertension – 2020

**Authors:** Leticia Costa Rebello, Marcos Christiano Lange, Rodrigo Bazan, Maramelia Miranda Alves, Gisele Sampaio Silva, Octavio Pontes

**Affiliations:** 1 Brasília DF Brazil Hospital de Base do Distrito Federal, Brasília, DF – Brazil; 2 Curitiba PR Brazil Universidade Federal do Paraná, Curitiba, PR – Brazil; 3 Botucatu SP Brazil Universidade Estadual Paulista Júlio de Mesquita Filho – Faculdade de Medicina Campus de Botucatu, Botucatu, SP – Brazil; 4 São Paulo SP Brazil Universidade Federal de São Paulo - Escola Paulista de Medicina, São Paulo, SP – Brazil; 5 Ribeirão Preto SP Brazil Universidade de São Paulo - Hospital das Clinicas da Faculdade de Medicina de Ribeirão Preto, Ribeirão Preto, SP – Brazil

**Keywords:** Hypertension, Intracranial Hemorrhage, Isquemic Stroke, Risk Factors, Mortality, Acute Treatment, Blood Pressure

**Dear Editor**

Initially, the Brazilian Society of Cerebrovascular Diseases (SBDCV) congratulates the Department of Arterial Hypertension of the Brazilian Societies of Cardiology, Hypertension, and Nephrology for the Brazilian Guidelines on Arterial Hypertension 2020 publication.^1^Our aim is to raise some issues related to stroke care and BP management in acute setting, an important topic of discussion and controversy. The guideline correctly attributes arterial hypertension as the main cause of ischemic stroke and intracranial hemorrhage. We would like to point out some issues regarding managing blood pressure (BP) in these patients.

Regarding BP control in patients with intracranial hemorrhage (ICH), the guidelines mentioned that “robust studies suggest that reducing BP (within 6h) to values <140/90 mmHg does not decrease important primary events, including mortality” (item 10.6.1), according to INTERACT-2 study.^2^The physiological response of increased BP levels in ICH is correlated with worse prognosis and hematoma expansion, as demonstrated in the INTERACT-1 study.^3^Subsequently, the INTERACT-2 trial compared intensive BP control [target systolic blood pressure (SBP) <140 mmHg] versus the guidelines-recommended levels (SBP <180 mmHg) in acute setting, and the primary outcome of death or functional dependence (modified Rankin Scale, mRS: 0-3 versus 4-6) were similar (55.6% in conventional treatment versus 52% with aggressive BP treatment, p=0.06).^2^Further, there was no significant difference in the shift distribution pattern in mRS. However, the ordinal analysis revealed a lower disability (mRS 0-2) with intensive BP treatment, with an odds ratio of 0.87 (95% confidence interval, 0.771.00; p=0.04), and additional better physical and mental quality, measured by EQ-5D scale.^2^Contrary to the 2020 Brazilian Arterial Hypertension Guidelines recommendation, and based on the results of this ordinal analysis, the current recommendation of the American Heart and American Stroke Association,^4^endorsed by the SBDCV, is to achieve an acute reduction of SBP in patients with ICH who present with high SBP (150-220 mmHg) without contraindications for intensive BP control. The SBP target is <140 mmHg, which can improve functional clinical outcomes. There is no sufficient data to systematically support the safety and effectiveness of the acute management of BP in patients with SBP >220 mmHg; however, a more aggressive reduction of BP in this profile of patients is reasonable, using intravenous drugs, dose titration and strict BP control in the acute phase.^4^Based on this trial and the recommendation of the societies mentioned above, we would like to suggest the correction of items 10.6.1 and 13.7 of the brazilian hypertension guideline, that states no benefit in reducing severe disability with intensive BP control.^1^We also emphasize that the proposed reduction in BP is safe.^2 – 4^The SBDCV does not recommend the proposed target of SBP <180 mmHg for acute ICH management.

Regarding the management of BP in acute ischemic stroke (IS), topic 10.6.2, we emphasize that BP reduction in patients who are candidates for thrombolysis should be performed when the values are >185/110 mm Hg in the first hour. After the end of thrombolysis, the recommended BP value is <180/105 mmHg in the first 24 h, as indicated in the hypertension guideline.^1^

Regarding topic 13.7.1, the recommended BP for the indication of thrombolytic treatment is <185/110 mmHg, and intravenous antihypertensive medication should be started immediately above this level. A contraindication to thrombolysis occurs only if refractory elevated BP occurs in three consecutive measurements, with an interval of 5 min, despite optimized treatment.

We are also unaware of the reference that suggests immediate BP reduction in patients with a transient ischemic attack (TIA), as suggested in Table 10.2.^1^In contrast, TIA is considered an equivalent to acute IS, and must be managed with the same parameters of a non-thrombolysed IS, or a IS not submitted to thrombectomy, that is, BP tolerability up to 220/120mmHg and suspension of oral antihypertensive drugs in the hyperacute phase of care, unless there are other impeding cardiovascular conditions to allow these blood pressure levels (e.g., acute myocardial infarction, aneurysm, or aortic dissection).^5^Thus, it is also worth reviewing the topic and table of recommendations for immediate BP reduction in TIA, as well as no reduction of BP in all types of IS (Table 10.2).

The denomination of stroke in our country is quite diverse, depending on the state or region of the country (use of terms “AVC - Acidente Vascular Cerebral” and AVE - Acidente Vascular Encefálico”), a fact that has been demonstrated in a Brazilian study.^6^For this reason, recently, the portuguese term “AVC” have been widely recommended by specialists in the field, patient organizations, together with the SBDCV and the Scientific Department of Cerebrovascular Diseases of the Brazilian Academy of Neurology, as well as in academic research, campaigns, educational activities, press releases and interviews, with the purpose of better educate the population regarding the disease, and to avoid using other terms that may confuse and hinder the rapid recognition, essential for immediate stroke treatment and better prognosis. Thus, for future hypertension guidelines, we suggest the use of this recommended standardized portuguese terminology: “AVC - Acidente Vascular Cerebral”, instead of other terms, like “AVE”.

Finally, we are grateful for the opportunity to present our observations. On behalf of the Brazilian Society of Cerebrovascular Diseases and the Scientific Department of Cerebrovascular Diseases of the Brazilian Academy of Neurology, our goal as a stroke society is to help and contribute with other partners in future discussions of topics involving managing patients with stroke.
