# Supplementary material for: Carta ao Editor Referente às Diretrizes Brasileiras de Hipertensão Arterial – 2020
Source: Arq Bras Cardiol. 2022 Jul 7;119(1):139–42. [Article in Portuguese] doi: 10.36660/abc.20210873 (PMC9352129; doi:10.36660/abc.20210873)
Supplement: Related texts

# Reply: Carta-resposta

**Authors:** Comitê Diretivo do DBHA2020, Weimar K Sebba Barroso, Cibele Isaac Saad Rodrigues, Luiz Aparecido Bortolotto, Marco Antônio Mota-Gomes

Carta resposta à Sociedade Brasileira de Doenças Cerebrovasculares e ao Departamento Científico de Doenças Cerebrovasculares da Academia Brasileira de Neurologia.

Inicialmente manifestamos nosso respeito e agradecimentos pela leitura atenta às Diretrizes Brasileiras de Hipertensão Arterial 2020 (DBHA2020),^1^e pelos comentários que permeiam o conteúdo exposto no capítulo 10, mais especificamente ao tópico 10.6 que trata da temática referente à hipertensão arterial (HA) e acidente vascular encefálico (AVE) hemorrágico e isquêmico.

Importante salientar que a DBHA2020^1^contou com a participação intelectual coletiva da Sociedade Brasileira de Cardiologia por meio do seu Departamento de Hipertensão Arterial, da Sociedade Brasileira de Hipertensão e da Sociedade Brasileira de Nefrologia, representadas por 97 especialistas nomeados por critérios científicos. Durante todo o ano de 2020, esses profissionais trabalharam com o objetivo comum de construir esse documento, elaborado a partir de reuniões semanais do comitê diretivo, duas reuniões com os 18 coordenadores de capítulo, e duas reuniões plenárias com os integrantes da DBHA2020. O texto final representa a opinião da maioria desse colegiado.

Passamos agora a discutir sobre os pontos questionados pelos subscritores da carta ao editor e, para isso, gostaríamos de tecer as seguintes reflexões:

A questão da meta de tratamento da HA, quando iniciar e quais medicamentos utilizar em eventos cerebrovasculares é certamente matéria de grande complexidade e um desafio, mesmo para especialistas.

Em janeiro de 2021, uma revisão narrativa compilou os principais estudos em AVE isquêmicos e hemorrágicos, apontando para um conservadorismo em relação à fase aguda desses eventos, e limitações, instransponíveis até o presente momento, mesmo quando se utilizam estratégias de revisões sistemáticas e metanálises.^2^

Sobre o Item 10.6.1 da DBHA2020,^1^que apresenta a seguinte redação sobre o tratamento da HA na fase aguda do AVE hemorrágico: “ *Se houver aumento da pressão arterial, podem ocorrer maior probabilidade de expansão do hematoma, aumento do risco de morte e pior prognóstico. Estudos robustos sugerem que reduzir a pressão arterial (dentro de 6 h) para valores <140/90 mmHg não diminuiu eventos primários importantes, inclusive mortalidade. Assim, não deve ser recomendada imediata redução da pressão arterial nos casos de AVE hemorrágico, a menos que o valor da pressão arterial sistólica esteja > 220 mmHg”.* Reforçamos que o texto afirma não haver evidência de redução de eventos primários, o que está em acordo com a carta encaminhada ao editor pela Sociedade Brasileira de Doenças Cerebrovasculares e pelo Departamento Científico de Doenças Cerebrovasculares da Academia Brasileira de Neurologia onde está escrito: “ *o estudo de fase 3 INTERACT-2 avaliou o controle intensivo de pressão arterial nestes pacientes, com meta pressão arterial sistólica <140mmHg versus controle de diretrizes da época (meta de pressão arterial sistólica <180mmHg). Analisando o desfecho primário de morte ou dependência funcional (modified Rankin Scale – mRS: 0-3), não foi alcançada significância estatística em relação à redução de pressão arterial na fase aguda para esse desfecho.”*

A nosso ver, assumir recomendação de diretrizes com base em resultados delineados como objetivos secundários, quando o objetivo primário não foi alcançado, não é a orientação mais adequada. A proposição no Quadro 10.2 para a redução da pressão arterial na fase aguda (dentro de 6 horas) após um acidente vascular encefálico hemorrágico atendeu à decisão da coordenação geral, coordenação do capítulo e da maioria em reunião plenária como a mais alinhada com as evidências científicas atuais.

Quanto à orientação de conduta na fase aguda do AVE isquêmico (item 10.6.2), a recomendação da DBHA2020 é para a redução da pressão arterial sistólica a valores menores que 180 mmHg, e da diastólica a valores inferiores a 105 mmHg apenas nos pacientes candidatos à trombólise, sem evidência de benefício clínico nos demais pacientes (Quadro 10.2). Pela leitura atenta que fizemos da Carta ao Editor, entendemos haver concordância com essa orientação que, coincidentemente, é a mesma do recém-publicado *Guideline da European Stroke Organization* (ESO),^3^e da *American Heart Association e American Stroke Association* .^4^

Em relação ao comentário sobre a conduta na fase aguda após acidente isquêmico transitório, estamos alinhados com as recomendações de outras sociedades científicas como, por exemplo, a última diretriz europeia de hipertensão arterial publicada em 2018 que cita na página 3086 a mesma orientação.^5^

No *guideline* da ESO,^3^cujos 11 autores são da área de neurologia, a primeira frase expressa exatamente a possibilidade construtiva de divergências: *“The optimal blood pressure (BP) management in acute ischaemic stroke (AIS) and acute intracerebral haemorrhage (ICH) remains controversial.”* Conclui o documento apontando para a necessidade de estudos randomizados e controlados que possam corroborar metas, tempo e estratégias para diminuição da pressão arterial na fase aguda em diferentes subgrupos de pacientes com AVE.

Finalmente, sobre a opção de nomenclatura utilizada nas DBH2020^1^– AVE –, essa foi a opinião da maioria dos integrantes como sendo o mais adequado a ser empregado no documento atual. Entendemos tratar-se de uma questão semântica e não anatômica propriamente dita e que a doença cerebrovascular pode envolver qualquer estrutura encefálica. É curioso que o descritor acidente vascular cerebral tenha 17 (dezessete) alternativas de sinonímia reconhecidas na Biblioteca Virtual de Saúde (BVS), entre elas, acidente vascular encefálico e sua correspondente abreviatura, AVE.^6^No entanto, essa sugestão será considerada na edição de uma nova atualização das diretrizes, tendo em vista os argumentos apresentados e a familiaridade existente na classe médica em geral com o termo AVC.

Uma vez mais, agradecemos a oportunidade de debate técnico e intelectual, e esperamos ter justificado os questionamentos encaminhados.

## References

[1] Barroso WKS, Rodrigues CIS, Bortolotto LA, Mota-Gomes MA, Brandão AA, Feitosa ADM, et al. Brazilian Guidelines of Hypertension - 2020. Arq Bras Cardiol. 2021;116(3):516-58. doi: 10.36660/abc.20201238.10.36660/abc.20201238PMC994973033909761

[2] Cantone M, Lanza G, Puglisi V, Vinciguerra L, Mandelli J, Fisicaro F, et al. Hypertensive Crisis in Acute Cerebrovascular Diseases Presenting at the Emergency Department: A Narrative Review. Brain Sci. 2021;11(1):70. doi: 10.3390/brainsci11010070.10.3390/brainsci11010070PMC782566833430236

[3] Sandset EC, Anderson CS, Bath PM, Christensen H, Fischer U, Gąsecki D, et al. European Stroke Organisation (ESO) Guidelines on Blood Pressure Management in Acute Ischaemic Stroke and Intracerebral Haemorrhage. Eur Stroke J. 2021;6(2):II. doi: 10.1177/23969873211026998.10.1177/23969873211026998PMC837006734780579

[4] Powers WJ, Rabinstein AA, Ackerson T, Adeoye OM, Bambakidis NC, Becker K, et al. Guidelines for the Early Management of Patients with Acute Ischemic Stroke: 2019 Update to the 2018 Guidelines for the Early Management of Acute Ischemic Stroke: A Guideline for Healthcare Professionals from the American Heart Association/American Stroke Association. Stroke. 2019;50(12):e344-e418. doi: 10.1161/STR.0000000000000211.10.1161/STR.000000000000021131662037

[5] Williams B, Mancia G, Spiering W, Rosei EA, Azizi M, Burnier M, et al. 2018 ESC/ESH Guidelines for the Management of Arterial Hypertension. Eur Heart J. 2018;39(33):3021-104. doi: 10.1093/eurheartj/ehy339.10.1093/eurheartj/ehy33930165516

[6] Health Sciences Descriptors: DeCS [Internet]. São Paulo (SP): BIREME/PAHO/WHO; 2017 – Stroke; [cited 2021 Dec 4]. Available from: https://decs.bvsalud.org/ths?filter=ths_termall&q=stroke

# Reply

**Authors:** Comitê Diretivo do DBHA2020, Weimar K Sebba Barroso, Cibele Isaac Saad Rodrigues, Luiz Aparecido Bortolotto, Marco Antônio Mota-Gomes

Response to the Brazilian Society for Cerebrovascular Diseases and the Scientific Department on Cerebrovascular Diseases of the Brazilian Academy of Neurology.

First, we would like to express our respect and gratitude to the reviewers for the careful reading of the 2020 Brazilian Guidelines on Arterial Hypertension (DBHA2020)^1^and for the comments on Chapter 10, more specifically on the topic 10.6 that addresses arterial hypertension (AH), and hemorrhagic and ischemic stroke.

It is worth pointing out that the DBHA2020^1^was developed by the collective effort of the Brazilian Society of Cardiology through the Department of Arterial Hypertension, the Brazilian Society of Hypertension, and the Brazilian Society of Nephrology, represented by 97 specialists chosen by scientific criteria. During the entire year of 2020, these experts worked together to construct this document, which was achieved by weekly meetings of the directive committee, two meetings with 18 chapter coordinators, and two plenary sessions of all members involved in the elaboration of the DBHA2020. The final text represents the opinion of the majority of this work group.

We will now discuss the questions raised by the authors of the Letter to the Editor, by first making the following considerations:

The question about the treatment target for HA, the time when treatment should be initiated, and the drugs of choice in cerebrovascular events is certainly a complex and challenging issue.

In January 2021, a narrative review summarized the main studies on ischemic and hemorrhagic stroke and pointed to a conservative approach in the acute phase, in addition to associated limitations that cannot be overcome even when strategies proposed by systematic reviews and meta-analyses are used.^2^

Regarding the item 10.6.1 of the DBHA2020,^1^which describes recommendations on the treatment of AH in the acute phase of hemorrhagic stroke: “In case of an increase in blood pressure, the odds of hematoma expansion, death and worse prognosis may increase. Robust studies have suggested that the reduction of blood pressure (within six hours) to levels < 140/90mmHg does not reduce important primary events, including mortality. Therefore, the immediate reduction of blood pressure in case of hemorrhagic stroke is not recommended, unless systolic blood pressure is > 220 mmHg ”. We reinforce that the text states that there is no evidence of reduction of primary events, which is in accordance with the Letter to the Editor submitted by the Brazilian Society of Cerebrovascular Diseases and by the Scientific Department of Cerebrovascular Diseases of the Brazilian Academy of Neurology, which affirms: “the phase 3 INTERACT-2 study evaluated the strict control of blood pressure in these patients, with a target systolic blood pressure below 140 mmHg *versus* the target recommended by guidelines (<180mmHg). Considering the primary outcome of death or functional dependence (modified Rankin Scale – mRS: 0-3), no statistically significance was found in this outcome by reducing blood pressure levels in the acute phase”.

In our opinion, it is not appropriate to adopt guidelines’ recommendations based on results obtained from secondary objectives, when the primary objective was not achieved. In Chart 10.2, the suggestion of reducing blood pressure (within six hours) in the acute phase after a hemorrhagic stroke was in line with the decision of the general coordination, chapter coordination and the plenary session, that this approach was in accordance with current scientific evidence.

As for the acute phase of ischemic stroke (item 10.6.2), the recommendation of the DBHA2020 is to reduce systolic blood pressure to values below 180 mmHg and diastolic blood pressure to values below 105 mmHg only for patients candidate for thrombolysis, with no evidence of clinical benefit for other patients. (Chart 10.2). Based on the close reading of the Letter to the Editor, we understand that there is a consensus on this recommendation, which coincidently is the same of the recently published European Stroke Organization (ESO), guidelines^3^and of the American Heart Association and American Stroke Association.^4^

In response to the comment on the management in the acute phase following a transient ischemic attack, we are in line with recommendations of other scientific societies, like the last European guidelines on arterial hypertension published in 2018 which recommends the same approach (see page 3086).^5^

In the ESO guidelines,^3^whose 11 authors work in the field of Neurology, the first sentence expresses the constructive possibility of divergence: “The optimal blood pressure (BP) management in acute ischemic stroke (AIS) and acute intracerebral hemorrhage (ICH) remains controversial”. The document concludes that further randomized controlled studies are needed to support treatment targets, time and strategies to reduce blood pressure levels in the acute phase in different subgroups of patients with stroke.^6^

Finally, concerning the nomenclature used in the DBH2020^1^– stroke – it was considered by most specialists as the most appropriate to be used in the current document. We understand that this is a semantic rather than an anatomic issue, and that cerebrovascular disease may involve any encephalic structure. Curiously, the descriptor brain vascular accident has 28 alternative synonyms, including its abbreviation, BVA, recognized by the virtual health library.^6^However, this suggestion should be further discussed in a new update of the guideline, because of the arguments presented, and the familiarity with the term stroke by physicians in general.

Once again, we are grateful for the opportunity to have this technical and intellectual debate, and we hope that we have clarified the questions raised.
